# Implementation frameworks for polypharmacy management within healthcare organisations: a scoping review

**DOI:** 10.1007/s11096-023-01534-8

**Published:** 2023-01-31

**Authors:** S. Al Bulushi, T. McIntosh, A. Grant, D. Stewart, S. Cunningham

**Affiliations:** 1 School of Pharmacy and Life Sciences, Robert Gordon University, Aberdeen, UK; 2 Ministry of Health, Muscat, Oman; 3 School of Nursing, Midwifery and Paramedic Practice, Robert Gordon University, Aberdeen, UK; 4 College of Pharmacy, Qatar University, Doha, Qatar

**Keywords:** Implementation framework, Organisational change, Polypharmacy management, Strategic framework

## Abstract

**Background:**

Several guidelines support polypharmacy management in individual patients. More organisational-level focus is needed on the use of implementation frameworks.

**Aim:**

To characterise the peer reviewed literature on implementation frameworks, focussing on barriers and facilitators to implementation at organisational level in the context of polypharmacy management.

**Method:**

A scoping review protocol was devised, supporting retrieval of studies published in English, reporting from any sector of practice. Medline, International Pharmaceutical Abstracts, Cumulative Index of Nursing and Allied Health Literature and Business Source Complete were searched to January 2022 using Medical Subject Headings including: ‘polypharmacy’, ‘deprescriptions’, ‘strategic planning’ and ‘organizational innovation’. A narrative approach to data synthesis was applied. Searching, data extraction and synthesis were undertaken independently by two reviewers.

**Results:**

After screening 797 records eight papers remained. Two were descriptive outlining details of specific initiatives, six used qualitative methods to explore determinants for implementation including barriers and enablers. Organisation level barriers included: poor organisational culture with a lack of sense of urgency and national plans, resource availability and communication issues including patient information and at transitions of care. Organisational facilitators included availability of government funding and regulatory environment promoting patient safety, a national emphasis on quality of care for older adults, co-ordinated national efforts and local evidence.

**Conclusion:**

Limited literature focusses on the use of implementation frameworks at organisational levels. This review highlights the need for further work on implementation frameworks in this context to help achieve effective organisational change.

## Impact statements

- This scoping review highlights the importance of developing and using structured implementation frameworks and organizational change elements for polypharmacy management.
- To contribute to safe, effective and economic patient care more research is needed on the use of organisational-level implementation frameworks for polypharmacy management.
- A focus on local contexts and cultures should be considered to a greater extent to facilitate the development of implementation frameworks for organisation change.
- More research is required on the impact of implementation frameworks and organisation change theory in the polypharmacy management context.

## Introduction

The world’s population is ageing with associated increases in multimorbidity [1, 2] which can result in a rise in medication burden and hence polypharmacy [3]. The prevalence of polypharmacy around the world varies by country, ranging from 24.3% in Europe [4] to 40% in the USA [5] and is even higher in the Middle East [3, 6, 7]. Aitken and Gorokhovich reported that the global health expenditure could be reduced by 0.3% through managing inappropriate polypharmacy [8]. A systematic review and meta-analysis of observational studies examined adverse health outcomes and health care utilisation outcomes of polypharmacy in older adults and found consistent evidence for inappropriate prescribing and hospitalisation [9]. Previous European and North American studies suggest that polypharmacy is a complex global issue [10, 11].

There has been much focus on the development of clinical guidelines and recommendations for the management of inappropriate polypharmacy. A clinical guideline has been defined as ‘… recommendations on how healthcare and other professionals should care for people with specific conditions. The recommendations are based on the best available evidence.’ [12]. As such, guidelines are often focussed on the process of individualising patient care based on the best evidence and often do not consider the wider organisational context and implications for healthcare management.

A review of polypharmacy management guidance [13] identified that only five European Union (EU) countries had produced guidance documents focussing specifically on polypharmacy management in older people and few existed in other parts of the world. Only the Scottish Government Model of Care Polypharmacy Working Group Polypharmacy Guidance included consideration of the importance of inclusion of change management strategies that matches organisational contexts to ensure identification of all aspects relevant to effective implementation including barriers and facilitators.

Indeed, in 2017 the World Health Organization (WHO) addressed polypharmacy in the third Global Patient Safety Challenge “Medication without harm” [14] and advised countries to consider implementation of organisational change management strategies to overcome barriers to executing polypharmacy management programs [15]. The EU-funded project ‘Stimulating Innovative Management of Polypharmacy and Adherence in the Elderly’ (SIMPATHY) [16] highlighted the importance of theory-based guideline implementation frameworks for organisational change management [17].

Implementing guidelines effectively into practice is complex and challenging and Kitson et al. proposed a ‘conceptual framework’. They posited that there is often a predominant focus on the level and nature of evidence for guideline implementation and identified a requirement for an equal focus on other aspects such as the context and environment, the actual process of implementation and its facilitation [18]. More recently, Moullin et al. have published a systematic review of implementation frameworks for innovations in healthcare and have proposed a ‘Generic Implementation Framework’. This outlines core implementation concepts including the need for comprehensive information on: process of implementation (steps/stages), characteristics of the innovation, definition of the context, barriers and enablers and strategies for evaluation [19].

Having polypharmacy management guidelines that outline approaches for individual patient care can be of limited value [13]. Consideration of implementation frameworks incorporating polypharmacy management at organisational levels are therefore considered of prime importance as part of global concerted efforts to improve appropriate use of medicines [14]. Although there are numerous studies on the use of polypharmacy management strategies at the patient level, there is a paucity of information about the effective use of implementation frameworks for polypharmacy management at the healthcare organisational level.

It has been noted that social context can be a key facilitator of quality improvement and that there are structural levels within socio-institutional theory including: macro—the system level, meso—the organisation level and micro—the team or individual level [20].

Fulop and Roberts have defined the meso-structural level of the ‘organisation’ in healthcare as ‘health care entities at any level providing any kind of health care’ [20]. This is distinct from the health system level which encompasses aspects external to the organisational entities and which may include, for example, government level health and social care units such as ministries or governmental departments. Fulop and Robert also assert that the majority of factors influencing quality improvement success relate to the meso-structural organisation level and include: ‘leadership, cultures, climate, organisational experience of quality improvement, organisational size, financial and clinical performance, data and information systems, knowledge and training’ [20]. For these reasons this review focusses on the ‘organisational level’.

### Aim

The aim of this review was to characterise the peer reviewed literature on implementation frameworks, with a focus on barriers and facilitators to implementation at organisational level in the context of polypharmacy management. It was designed to address the following questions (1) what are the characteristics of the literature including study aims, research designs, methods and study populations ? (2) what are the different characteristics of these frameworks, including the process of their development, structure and content, evaluation/monitoring and assessment of implementation outcomes? (3) what are the reported barriers and facilitators that influence the use of implementation frameworks for organisational change?

## Method

A scoping review is often used when a research topic has not been widely explored and summarised through previous reviews [21]. They are useful for exploring topic areas where there is ambiguity around definitions and concepts which would make the use of other methods such as systematic review methodology challenging [22]. This scoping review followed the 6-stage methodological framework of Arksey and O’Malley to inform the conduct of the review [23]. Reporting was guided by the Preferred Reporting for Systematic and Meta-Analysis (PRISMA) extension for scoping reviews [24] and related guidance [25].

### Eligibility criteria

Eligibility criteria and their rationale for inclusion and exclusion of studies in the review are presented in Table 1. This includes the detail of the population, concept and context of this review. The defined populations, concepts and contexts were broad to ensure consideration and inclusion of a wide range of literature.

**Table 1:** Eligibility criteria and search terms for study selection

Eligibility criteria for study selection	
Inclusion criteria	Rationale for inclusion and exclusion
Population: all healthcare organisations, professions and other stakeholders including patients/carers	
Concept: Use of implementation frameworks for organisational change regarding polypharmacy management	
Context: Country: Organisations within all countries (global) Sector: All sectors of practice and specialties, all levels of care: primary/secondary/tertiary within organisations	All relevant areas and settings were included to ensure inclusion of a broad base of global literature
Language: English	
Data range: No restriction on research dates	
Types of literature: Full text peer reviewed papers reporting empirical data from primary research, descriptive articles, review articles including systematic reviews/scoping reviews/narrative reviews	Grey literature, conference abstracts, protocols, book reviews, opinion articles and editorial reviews were excluded
Search terms	
Polypharmacy OR Prescribing: Polypharmacy management OR Polypharmacy (MH) OR Inappropriate polypharmacy OR Multiple medication* OR Comorbidity (MH) OR Deprescriptions (MH) AND Rational prescribing OR Prescribe* OR Prescribing error OR Inappropriate prescribing (MH) OR Therapeutics (MH) OR Drug prescriptions (MH) OR Drug Overdose (MH) OR Prescription drug overuse (MH) OR Prescription drug misuse (MH) OR Prescription drug diversion (MH)	Framework OR Organisational change: Framework OR Strategic planning (MH) OR Strategic framework OR Organisational change OR Organizational change OR Organizational innovation (MH) OR Change management (MH) OR Organizational objectives (MH) OR Organizational policy (MH) OR Organizational culture (MH) OR Organizational affiliation (MH) OR Organizational models (MH)

MH = MeSH Heading

### Information sources

The following electronic databases were searched; Medline, International Pharmaceutical Abstract, Cumulative Index to Nursing and Allied Health Literature and Business Source Complete. No restriction was made on search dates with searches from database inception to January 2022.

### Search strategy

Relevant keywords and Medical Subject Headings (MeSH) terms were used including: ‘polypharmacy’, ‘deprescriptions’, ‘strategic planning’ and ‘organizational innovation’. Combination of search terms, Boolean operators (such as OR, AND), and truncations (*) were used as appropriate to broaden the search and to retrieve all relevant papers (Table 1).

### Literature source selection

To identify relevant papers, a wide range of literature was considered: full text peer reviewed papers reporting primary research, and descriptive and review articles. Grey literature, conference abstracts, protocols, book reviews, opinion articles, and editorials were excluded. Two researchers searched the databases independently using the agreed search strategy and cross-checked findings. All relevant studies were imported to RefWorks and duplicates removed. Initially two researchers independently screened the titles and abstracts of all retrieved papers against the pre-piloted inclusion criteria; papers were included if both reviewers agreed they should be. Full-text articles for all included papers were similarly reviewed. Any disagreements were adjudicated by a third member of the team.

### Data charting process

The data fields to be extracted from the selected studies were agreed by the research team based on the scoping review aims and questions. A data extraction tool was developed and reviewed by the research team then modified based on a piloting and subsequently used to extract data from the selected full-text articles. Data extraction was conducted independently by two researchers and discussed with the entire review team.

The following information was recorded: authors, year of publication, title, aim/objectives, methods, characteristics of the implementation strategies and frameworks, setting, country, sector, specialty, professions of the participants, intervention findings, and barriers and facilitators to implementation.

### Summary, synthesis, and reporting of results

The results of the scoping review are presented using a descriptive narrative approach to data synthesis.

## Results

### Selection of papers

**Fig. 1:**
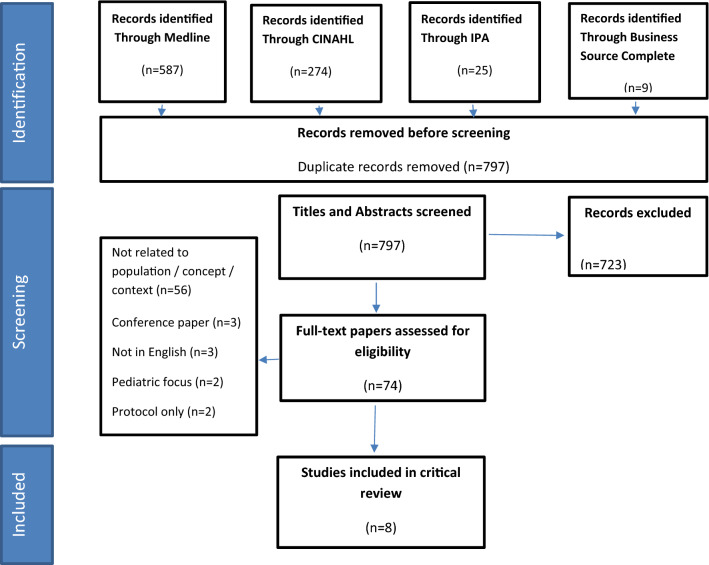
Search inclusion process PRISMA flow diagram

The PRISMA flow diagram in Fig. 1 provides information on the steps for selection of papers from the four databases. Of the 797 records identified after removal of duplicates, 723 were removed after title and abstract screening and a further 66 removed after full-text review. Eight remaining articles met the eligibility criteria.

Quantitative and textual summaries of the findings based on the extracted data are presented in Table 2.

**Table 2:** Data extraction table for included papers (N = 8)

Authors, publication year	Title	Methods	Characteristics of implementation strategies and frameworks	Setting, country, sector, specialty, professions participants, Intervention	Findings	Barrier and facilitators to implementation
McIntosh et al. [17]	A case study of polypharmacy management in nine European countries: Implications for change management and implementation	Desk review of published and grey literature on polypharmacy management: key informants interviewed stakeholders involved in policy development and experts involved in the development and implementation of polypharmacy initiatives as well as focus group of clinicians and managers Change management principles (e.g. Kotter) and normalization process theory (NPT) informed data collection and analysis	Locally adapted solutions, organisational culture supporting innovation and teamwork, adequate workforce training, multidisciplinary teams, changes in workflow, redefinition of roles and responsibilities of professionals, policies and legislation supporting the initiative, and data management and information and communication systems to assist development and implementation	Country: European Union countries Participants: Policy development stakeholders and implementation, clinicians and managers	Only five countries had well developed polypharmacy initiatives. Four had none Case study findings supported implementation of complex innovations. Analysis used Kotter’s principles and identified a need to create a sense of urgency and a strategic vision and that change management frameworks might be useful	Barriers: lack of data to create a sense of urgency, lack of use of change management frameworks Facilitators: local adaptation, positive organisational culture, redefinition of roles, policies and legislation, good data management and information and communication systems
Scott et al. [26]	Development of a hospital deprescribing implementation framework: a focus group study with geriatricians and pharmacists	Exploratory qualitative approach adopted to understand the barriers and enablers to deprescribing experienced and perceived by geriatricians and pharmacists, focus groups discussions, thematic analysis to explore barriers and enablers inductively. TDF used to identify Behaviour Change Techniques (BCTs) to address barriers and enablers. Topic guide designed to invite discussions about barriers and enablers to deprescribing	Hospital deprescribing implementation framework (hDIF) developed, matching identified theoretical domain framework (TDF) domains to 44 BCTs which can be selected according to local context developed through analysis of data from focus group discussions Not implemented so no evaluation/ monitoring	Country: United Kingdom Setting: Four hospitals across three English counties, two of which were 1,000 and 1,200 bed teaching hospitals with four and six geriatric wards, respectively, and two were 450 and 550 bed district general hospitals each with three geriatric wards Participants: 54-geriatricians and pharmacists representing four UK hospitals attended eight focus groups	The behavioral determinants and their associated intervention components provided a hDIF Four themes were identified: (i) role of different professionals (ii) the inpatient environment (iii) consideration of outcomes (iv) attitudes towards medicines Agreement that geriatricians should lead on de-prescribing supported by pharmacists	Barriers: patient and caregiver attachment to medication, perceptions deprescribing is riskier, pharmacists’ working patterns limiting capacity, low hospital priority) and incentives Facilitators: alignment of roles between pharmacists and geriatricians, confidence in their existing knowledge and capabilities to undertake their roles, patient dislike of medication, improved outcomes for patient’s, practitioners and hospital
Straßner et al. [27]	German healthcare professionals’ perspective on implementing recommendations about polypharmacy in general practice: a qualitative study	Qualitative approaches (semi-structured interviews and focus groups), used to identify determinants (both barriers and enablers) for the implementation. Principles of qualitative content analysis used for analysis. This gave two sets of categories which were assigned to the Tailoring Interventions for Chronic Diseases (TICD) checklist, a comprehensive framework of determinants of practice	The TICD was used which arose from an international cluster-randomised controlled trial conducted in five European countries. This considered tailored interventions to improve the care of multimorbid patients using polypharmacy	Country: Germany Setting: University Hospital Heidelberg and three hospitals near Heidelberg Participants: Interviews with 24 general practitioners (GPs), 4 other medical specialists, 1 pharmacist, working in inpatient and outpatient care and 3 nurses and 6 medical assistants as well as 2 mixed focus groups with 17 professionals were Conducted	Identification of 93 determinants related to all 7 main domains and to 25 of the 57 subdomains on the TICD checklist including guideline factors, patient factors, individual healthcare professional factors, social, political and legal factors, incentives and resources, and capacity for organisational change	Barriers: No barriers evident in the paper Facilitator: A proportion of the identified determinants were connected to pharmacological knowledge and so interventions to improve this may be effective
McNamara et al. 2017 [28]	Health professional perspectives on the management of multimorbidity and polypharmacy for older patients in Australia	Qualitative semi-structured interview study with 26 HCPs working in metropolitan and rural areas of Victoria and South Australia. The semi-structured interview was focused around six domains relating to current multimorbidity	The American Geriatrics Society (AGS) published Guiding Principles for the Care of Older Adults with Multimorbidity (2012) was used to define how the healthcare team should function to improve care	Country: Australia—Victoria and South Australia Setting: Primary settings Participants: 26 HCPs (14 prescribers and 12 practiced in primary care) were recruited from relevant medical, dentistry, nursing, pharmacy and allied health backgrounds, and almost half worked in primary care settings	HCPs perceived shared decision-making as important, but no participant made reference to exploring patient goals Prescribing HCPs questioned value of clinical guidelines when making decisions. All HCPs reported problems with incorporating patients’ prognoses and continuity of care. Hospital-based HCPs were considered more accessible due to collegiality	Barriers: Poor coordination of care with multiple prescribers resulting in complex regimens and difficulties acquiring information, delayed or vague discharge summaries, inaccurate medication lists from GPs. Time constraints and lack of responsibility taking Facilitators: Not adjusting medication in hospital may avoid errors and confusion Post-discharge review checks. GPs and pharmacists highlighted increased levels of government remuneration could help
Stewart et al. [29]	A modified Delphi study to determine the level of consensus across the European Union on the structures, processes and desired outcomes of the management of polypharmacy in older people	Three Delphi rounds conducted via email, with panel being given summative results and collated, anonymised comments at the commencement of each. Forty-six statements were developed on healthcare structures, processes and desired outcomes, with consensus defined at ≥ 80% agreement	This was a consensus-based study and as such it sought to explore consensus in relation to the Donabedian approach of structure, process and outcome. The focus of the statements included; strategic development, change management and indicator measure and awareness raising for implementation	Country: European Union Participants: Five members from each country (n = 28), giving 140 members The five included: one policy maker, two healthcare commissioners, one healthcare provider director level and one clinician (physician or pharmacist)	Consensus was obtained for statements relating to: potential gain arising from polypharmacy management (3/4 statements); strategic development (7/7); change management (5/7) indicator measures (4/6); legislation (0/3); awareness raising (5/5); polypharmacy reviews (5/7); and EU vision (0/7)	Not relevant for this paper
Conroy et al. [30]	Improving acute care for older people at scale - The Acute Frailty Network (AFN)	This descriptive paper covers different approaches used to achieve high quality care for frail older people with urgent care needs. The evidence base is summarized and the paper described how the Acute Frailty Network has set about delivering improvements at scale	The AFN was designed using the Breakthrough Series Collaborative (BTS) approach, involving health and social care systems working with support by national clinical and improvement experts and sharing experiences through national networking events. The BTS uses a specific quality improvement approach focusing on Plan-Do-Study-Act cycles to create change	Country: Portsmouth, United Kingdom	The paper covers what the Comprehensive Geriatric Assessment (CGA) is and indicate it is considered best practice. It also covered differences between CGA and normal acute care. Communication, sepsis, confusion, falls, polypharmacy and end of life care. Covers the Acute Frailty Network (AFN)	Barriers: Challenges doing a CGA in urgent care settings because of constraints of time and priority of urgent medical treatment Facilitators: Adoption of clinical professional standards to reduce variation and develop a measurement mind-set. Education and training for key staff and clinical change champions including an executive sponsor. Patient and public involvement
Alkema and Frey [31]	Implications of Translating Research into Practice: A Medication Management Intervention	This descriptive article describes in detail the Intervention as an example of translating an evidence-based practice beyond its original efficacy trial in a home healthcare program into a care management program. It outlines in detail the steps in implementation and an evaluation plan	Promoting Action on Research Implementation in Health Services framework (PARIHS) was used to explore critical factors involved in translating research into practice From results a Medication Management Model (MMM) and the implementation of the MMM is described	Country: California, United States of America Participants: Frail, community-dwelling, older adult population	The paper identified organizational variables relevant in assessing the feasibility of translating the MMM into the applied practice settings. The study suggests that although implementing research into practice can positively impact client care, professional skill enhancement and organizational effectiveness, it is very challenging	Barriers: Geographically and culturally diverse settings for implementation Operational differences across the implementing sites Facilitators: Nature of the client population of high-risk community dwelling older adults with co-morbid medical conditions Availability of government funding streams. The nature of the regulatory environment
Kouladjian O’Donnell et al. [32]	Development and dissemination of the national strategic action plan for reducing inappropriate polypharmacy in older Australians	Development of rNSAP: Written invitations to national stakeholders and ’face-to-face meeting to identify key priority areas for workshop. Dissemination: Delegates of a conference participated in a small group workshop tasked with identifying key contacts, describing the issue and its importance for stakeholders. Iinformation generated from the small groups were categorized. Then individualised letters were sent to each contact to garner views on the rSNAP	This work describes an approach to the development and dissemination of a national strategy for Quality of Medicine use that focusses on recommendation for a national strategic action plan to reduce polypharmacy. This was based on a previously published framework of system level strategies for facilitating deprescribing in practice	Country: Australia Participants: Initially multi-disciplinary professional groups, academia, quality use of medicines and aged care organisations, consumer groups and regulatory agencies in conjunction with members of Australian Deprescribing Network	Development: synthesis of national action plan on 7 key actions crossing all health system. Dissemination: Views from a total of 18 of the 99 organisations across Australia sent rNSAP. Wide range of responses and suggestions made e.g. need to review teaching curricula and circulating the report to teaching staff, need to develop resources, referral of the rNSAP to government leaders etc. Eight activities outlined and the progress made in relation to each	Barriers: None articulated within the paper Facilitators: Co-ordinated national effort and engagement of a wide group of stakeholders, dissemination of rNSAP in conjunction with practice change programmes

*AGS* American Geriatrics Society, *BCT* Behavior Change Technique, *CGA* Comprehensive Geriatric Assessment, *HCP* Health Care Professionals, *hDIF* Hospital deprescribing implementation framework, *MMM* Medication Management Model, *NPT* Normalization Process Theory, *PARIHS* Promoting Action on Research Implementation in Health Services framework, *rNSAP* Recommendations for a National Strategic Action Plan, *TDF* Theoretical Domains Framework, *TICD* Tailoring Interventions for Chronic Diseases

### Characteristics of the literature

Seven of the included studies were conducted between 2016 and 2020 [17, 26–30, 32]. Studies included addressed mainly the management of polypharmacy. They focused on: the availability of policies and guidelines on polypharmacy management in older people [26, 32]; the characteristics of healthcare professionals’ barriers and facilitators to polypharmacy management [26–28]; and the development of frameworks of interventions to facilitate the implementation and sustainability of polypharmacy management programs [17, 26, 29].

Two papers provided detailed descriptions of the characteristics of programs that addressed the care of frail older people but did not carry out data collection [30, 31]. In terms of research design, the other 6 papers [17, 26–29, 32] mainly used qualitative methods including: interviews [17, 27, 28], focus groups [17, 26, 27] and Delphi consensus [29] and workshops [32].

In relation to study populations five of the studies that were conducted in European countries [17, 26, 27, 29, 30]. One was conducted in the United States [31] and two in Australia [28, 32]. Three studies [17, 29, 32] were conducted at national levels with two of these related to the EU funded SIMPATHY project [17, 29] and the other conducted in conjunction with Australian Deprescribing Network [32]. Other papers focused on the healthcare organisational level of: primary care [28, 31], hospital inpatient only [26] and inpatient and outpatient care [27]. The two descriptive studies did not specify a focus but seemed to take a cross-sectoral approach [30, 31].

The two studies from the SIMPATHY project involved stakeholders (clinicians and managers) working in policy development and implementation [17, 29]. One paper involved professional groups, academia, aged care organisations and regulatory agencies [32]. Other involved geriatricians [26], pharmacists [26–28], general practitioners [27, 28], medical specialists [27], nurses [27, 28], and medical assistants [27].

### Synthesis of characteristics of implementation frameworks

Articles included different implementation strategies and frameworks for polypharmacy management which included details of development, structure and content, evaluation, monitoring, and assessment of implementation outcomes. Strategies suggested for effective change management included the Plan-Do-Study-Act (PDSA) [30], Kotter’s eight-step change model [17, 29], and Normalization Process Theory (NPT) to evaluate implementation processes [17]. Scott et al. used the Theoretical Domains Framework (TDF) to identify suitable behaviour change techniques to inform hospital de-prescribing frameworks for polypharmacy management [26]. The American Geriatrics Society (AGS) Guiding Principles for the Care of Older Adults with Multimorbidity was used by Australian researchers as a framework for analysis and informed the process of designing domains related to multimorbidity management [28] and Kouladjian O’Donnell et al. synthesised findings of a workshop into recommendations for an Australian national strategic action plan (rNSAP) to reduce inappropriate polypharmacy [32]. One study used a translational research framework, Promoting Action on Research Implementation in Health Services (PARIHS), as a tool to translate a medication management model into practice settings [31]. Straßner et al. used the ‘Tailoring Interventions for Chronic Diseases (TICD) checklist’, a comprehensive framework of determinants of practice to improve the care of patients with multimorbidity and polypharmacy. This approach was developed and evaluated in a cluster-randomised controlled trial, but little information was presented regarding the framework development and implementation [27].

### Synthesis of barriers and facilitators to implementation

The literature included in this review highlights the many barriers that can affect implementation of polypharmacy guidelines. Many of these were at the organisational-level and so very relevant to this review. However, others were at health system level, healthcare professional and individual patient levels and in view of the integrated nature of the review many are considered here.

Health system-level barriers included a lack of data which would facilitate the creation of a sense of urgency, the absence of national plans for implementation, monitoring, and evaluation, geographically and culturally diverse settings for implementation, and operational differences across implementing sites [17, 28, 30].

Barriers at the organisational level included: poor medication safety culture and resource availability [17, 26], system limitations, lack of availability of information for patients and transfer at transitions of care [26]. Poor communication systems were also highlighted as impeding implementation [17, 28] as did a lack of time for practice e.g. time for conducting structured medication reviews [27]. Organisational and healthcare system level facilitators included availability of government funding streams and strict regulatory environments, a national emphasis on quality of care for older adults and co-ordinated national efforts [17, 29, 30, 32].

Healthcare professional focussed barriers, many of which arose from organisational issues included: poor care coordination, lack of time, unclear allocation of tasks and responsibilities, lack of required competencies for pharmacists to make decisions, educational and training differences among staff, and a lack of shared decision making among healthcare professionals [27, 28, 30]. Barriers at the patient level were around social influences such as patient perceptions, expectations, and preferences in relation to medication [26]. Patient level facilitators included levels of patient access to healthcare resources, patient negative experiences of medications and perceptions around improved outcomes for patients [26].

## Discussion

### Statement of key findings

This review summarises literature on frameworks used and barriers and facilitators for their implementation for change, in relation to polypharmacy management, at organisational levels. The majority of articles were published between 2016 and 2020 with an array of geographic locations. Two were descriptive papers outlining details of specific initiatives, five papers used a range of qualitative methods to explore the rationale for the development of polypharmacy management initiatives and determinants for the implementation including barriers and enablers. Organisational level barriers included: poor organisational culture with a lack of sense of urgency and national plans, resource availability and communication issues including patient information and transition of care. Organisational facilitators included availability of government funding and regulatory environment promoting patient safety, a national emphasis on quality of care for older adults, co-ordinated national efforts and local evidence. This review shows that while polypharmacy management guidelines have been developed in some countries, there has been a limited focus on the development and implementation of frameworks, especially at the organisational level. There was limited literature for the use of implementation frameworks for polypharmacy management at the organisational level.

### Strengths and limitations

This is the first scoping review that synthesises literature related to organisational change in polypharmacy management. This review focussed on peer reviewed published literature and as such excluded grey literature, conference abstracts, protocols, book reviews, opinion articles and editorial reviews. In view of this, some papers may have been omitted but the authors felt this focus was important and appropriate given the aims of the review. The search strategy included two overarching terms relevant to the review aim along with a range of over 29 sub-terms. It is possible that other terms could have been included but the use of MESH® terms and explosion of subject headings within the search databases ensured comprehensive inclusion of paper for screening. This review included only articles published in English as there was no resource available for translation services. It is also acknowledged that there are many factors that can influence quality improvement in relation to medication burden. The review does not attempt to cover the many facets of quality medicines use of which polypharmacy is acknowledged to be only one.

### Interpretation

Common organisational contextual factors influencing the processes of quality improvement approaches were identified in papers in Kaplan et al’s systematic review, these included: organisational characteristics (e.g., size, ownership), leadership from top management, competition, organisational culture and data infrastructure/information systems [33]. These were considered to be broadly similar to constructs included in established theories of implementation and organisational change [34, 35].

Given this, an approach within implementation science to address such organisational factors is to consider how ‘frameworks’ can be used to help support innovation. Bauer et al. define ‘frameworks’ as ‘… a set of constructs that organise concepts and data … provide a prescriptive series of steps summarising how implementation should ideally be planned and carried out.’ [36]. Such frameworks offer a structured, robust method for operationalising and evaluating innovation.

Co-ordinated national efforts focussing on organisations and use of frameworks for implementation are highlighted from the studies by McIntosh et al. [17], McNamara et al. [28], Alkema and Frey [31] and Kouladjian et al. [32]. These show that factors that enable the implementation of polypharmacy strategies include: governmental support, availability of policies and legislations, funding, strict regulations and availability of good quality data. This is also supported by McIntosh et al. [37], Gennimata et al. [38], and Kempen [39] who highlighted a national focus on quality of care for older adults as well as the existence of health policies focusing on improved care for patients with complex chronic diseases. This is important since it has been shown that for implementation of evidence-based practice it is important to have a focus on the level and nature of evidence for guideline implementation along with an equal focus on other aspects such as the context and environment [18].

The papers by Conroy et al. [30] and Alkema and Frey [31] were descriptive in nature. Each outlined details of quite different projects with relevance to implementation of polypharmacy initiatives and offer vital information concerning this. Moullin et al. in their systematic review of implementation frameworks highlight the need to give consideration to: process of implementation (steps/stages), characteristics of the innovation, definition of the context, barriers and enablers and strategies for evaluation [19].

Despite the variety of methods used within included papers there was a clear message that polypharmacy continues to be considered an important issue that can result in improvements in the care of older people [30, 32] and have positive impacts through utilisation of change management frameworks at the organisational level [17, 31]. This is supported by Kempen et al. who have stated that ‘.organisational change strategy is a key factor involved in the implementation and sustainability of polypharmacy management programs’ [39].

The importance of consideration of context and environment within conceptual frameworks for implementation of initiatives has been highlighted [18]. Some papers highlighted the fact that challenges during the implementation process arose from barriers either at organisational level, including a lack of clear responsibilities for who conducts polypharmacy reviews, or within hospitals where initiatives were not considered as a priority [26, 30]. Similar barriers were demonstrated by Kempen et al. [39], who reported that a lack of attention when integrating new practices into daily workflows is also a barrier to implementing polypharmacy management programs. Gennimata [38] reported extreme financial pressure, a lack of organisational culture supporting multidisciplinary teams, and a lack of shared decision making and leadership from central health authorities as a barrier to implementation of polypharmacy management. The work by McIntosh et al. [17] and Stewart et al. [29] noted that without a fully coordinated strategy taking into consideration change management the desired outcomes are often not fully achieved and sustained across a population irrespective of the clarity and robustness of specific innovation.

Structured organisational change strategies are generally considered to enhance the data collection and implementation processes. These are entirely compatible with the principles of implementation frameworks where there is an expectation to clearly define aspects such as process of implementation, characteristics of the innovation, context and barriers and enablers [19]. Such organisational change strategies were evident in the included papers with the use of Kotter’s eight-step process used in the SIMPATHY project [29], Plan-Do-Study-Act quality improvement cycle approach by Conroy et al. [30], and the use by Alkema and Frey, of a translational research framework promoting action on research implementation in health services [31]. However, none of the studies specifically mentioned development and implementation of polypharmacy management frameworks.

The literature identified for inclusion in this review helps provide insights to the approaches taken to use structured approaches to address the introduction of polypharmacy management at organisational levels. Fulop and Robert proposed that there should be co-design and dissemination of tools, such as implementation frameworks [19] that enable organisational factors to be taken into account before beginning improvement interventions. In turn this would support relevant, contextualised intervention development which is systematically embedded within these implementation frameworks [20].

### Further research

Further research should focus on the development and testing of implementation frameworks at the meso-structural organisational level. Initial work could focus on definitions relating to concepts and contexts to facilitate cross-country and sector comparisons. This could be followed by consensus-based approaches for the development of context specific, theory-based implementation frameworks. Specific interventions to operationalise the frameworks could then be developed, implemented and evaluated with cognisance of the Medical Research Council guidance on developing and evaluating complex interventions [40].

## Conclusion

Although initiatives and guidelines for polypharmacy management are available, this review demonstrates that there is a lack of research focussed on implementation frameworks and how they can be used for change at organisational levels. Implementation of polypharmacy management programs are unlikely to fully achieve the desired outcomes unless implementation frameworks for organisational change are more fully considered.
